# Discovery of a novel and a rare Kristen rat sarcoma viral oncogene homolog (KRAS) gene mutation in colorectal cancer patients

**DOI:** 10.1080/21655979.2021.1960715

**Published:** 2021-08-08

**Authors:** Mahmood Rasool, Angel Carracedo, Abdulrahman Sibiany, Faten Al-Sayes, Sajjad Karim, Absarul Haque, Peter Natesan Pushparaj, Muhammad Asif, Niaz M. Achakzai

**Affiliations:** a Center of Excellence in Genomic Medicine Research, Faculty of Applied Medical Sciences, King Abdulaziz University, Jeddah, Saudi Arabia; b Department of Medical Laboratory Technology, Faculty of Applied Medical Sciences, King Abdulaziz University, Jeddah, Saudi Arabia; c Genomic Medicine Group, University of Santiago De Compostela, Spain; d Faculty of Medicine, KAUH, King Abdulaziz University, Jeddah, Saudi Arabia; e King Fahd Medical Research Center, Faculty of Applied Medical Sciences, King Abdulaziz University, Jeddah, Saudi Arabia; f ORIC, Department of Biotechnology, Buitems, Quetta, Pakistan; g Department of Molecular Biology, City Medical Complex, Kabul, Afghanistan; h Department of Molecular Biology, DNA Section, Legal Medicine Directorate, Ministry of Public Health, Kabul, Afghanistan

**Keywords:** *KRAS*, Colorectal cancer, Novel mutation, DNA sequencing, Saudi population

## Abstract

Colorectal cancer (CRC) is one of the most important causes of morbidity and mortality in the developed world and is gradually more frequent in the developing world including Saudi Arabia. According to the Saudi Cancer Registry report 2015, CRC is the most common cancer in men (14.9%) and the second most prevalent cancer. Oncogenic mutations in the *KRAS* gene play a central role in tumorigenesis and are mutated in 30–40% of all CRC patients. To explore the prevalence of *KRAS* gene mutations in the Saudi population, we collected 80 CRC tumor tissues and sequenced the *KRAS* gene using automated sequencing technologies. The chromatograms presented mutations in 26 patients (32.5%) in four different codons, that is, 12, 13, 17, and 31. Most of the mutations were identified in codon 12 in 16 patients (61.5% of all mutations). We identified a novel mutation c.51 G>A in codon 17, where serine was substituted by arginine (S17R) in four patients. We also identified a very rare mutation, c.91 G>A, in which glutamic acid was replaced by lysine (E31K) in three patients. In conclusion, our findings further the knowledge about *KRAS* mutations in different ethnic groups is indispensable to fully understand their role in the development and progression of CRC.

## GRAPHICAL ABSTRACT

**Figure:**
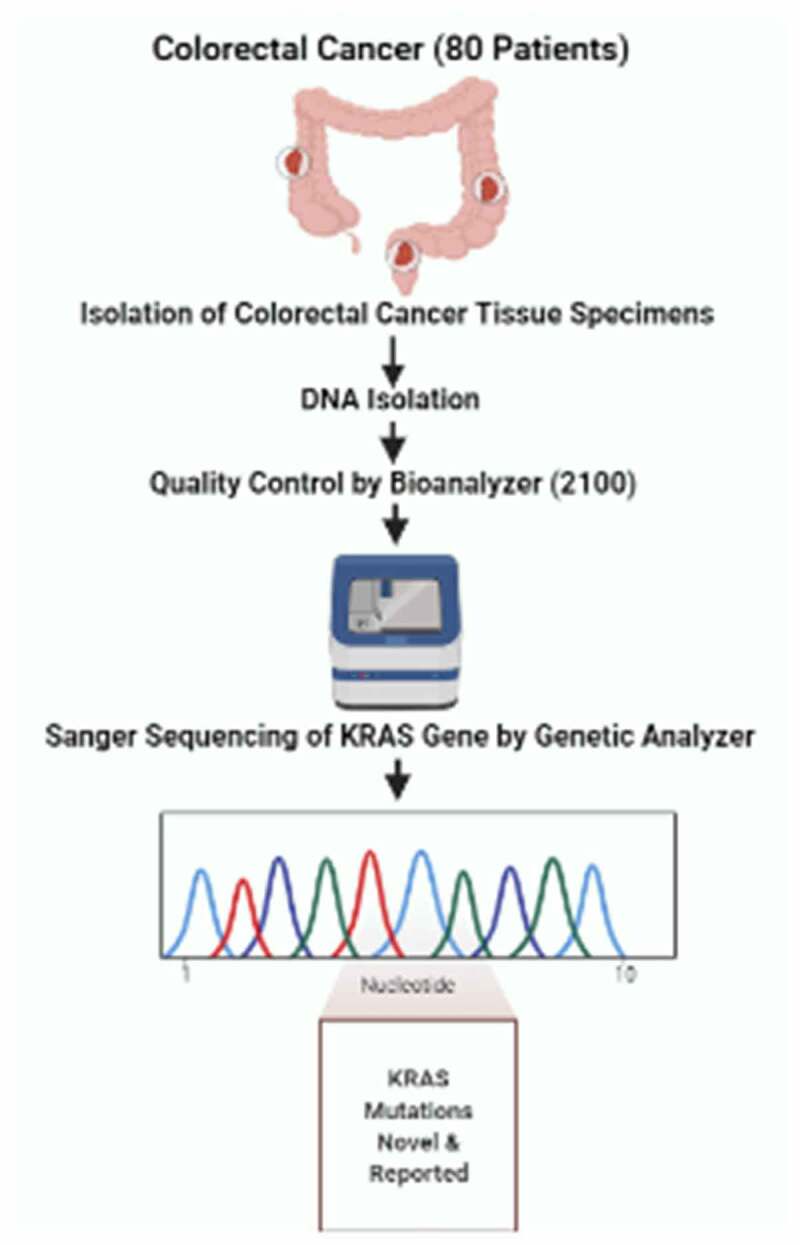

## Introduction

Colorectal cancer (CRC) is the second leading lethal malignancy and the third most common cancer diagnosed in both sexes globally [1,2]. Notably, nearly 20% of metastatic disease has been reported in CRC patients at the time of diagnosis [3]. According to colorectal statics 2020, an estimated 104,610 new cases of colon cancer are projected to be diagnosed in the United States [1,4]. Although most of these occur in adults aged 50 years and older as compared to individuals aged less than 50 years, 17,930 (12%) newly diagnosed cases of CRC were estimated [4]. It was reported that approximately 1.5 million of Americans are living with CRC till date and nearly 50,000 deaths occurs annually in the United States, the new data shows alarming leap in CRC in both newly diagnosed cases and mortality rates. The number of newly diagnosed CRC individuals was estimated to be 147,950, and deaths due to this disease were reported to be nearly 53,200, including 3640 decedents (7%) with individuals younger than 50 years in 2020 in the United States in 2020 [1,4].

Notably, CRC in Saudi Arabia is also reported to be the third leading cause of death in individuals aged less than 70 years according to the World Health Organization (WHO) estimation data among 22 countries also predicted that one in eight deaths reported in the Kingdom of Saudi Arabia is due to CRC malignancies [5–7]. In Saudi Arabia, one-third of CRC cases are diagnosed with distant metastases; as a result, this stage is considered a major contributor to premature death among the Saudi population [8]. However, according to the latest Saudi National Cancer Registry data, CRC remains at the same position in terms of malignancies, but the increasing trend in the incidence of CRC is alarming, as evident from the 2014 data, where 2047 newly diagnosed CRC cases were reported [7]. Similarly, previous epidemiological trends also support the increasing incidence, as evidenced by the latest observation where CRC accounted for 10.4% of all newly diagnosed cases with a male to female ratio as compared to the previous report of 2004, where 9.3% of new CRC cases were reported [9,10]. Notably, the age-standardized rate (ASR) of CRC in Saudi Arabia has also increased significantly, as indicated by the fact that (overall, female, and male) ASR of 7.3, 6.3, and 8.3 per 100,000 population, respectively, in 2004 to 9.6, 9.2, and 9.9/100,000, respectively [9,11]. According to the estimation by the General Authority for Statistics of Saudi Arabia, as the total KSA population was estimated to be 20,768,627 in 2018 (49% female and 51% male), and they are primarily young and living mostly in the central (22.83%), western (22.13%), and eastern regions (15.39%) of Saudi Arabia. Interestingly, nearly half of the population is under 25 years of age, and 35% between 20 and 39 years of age [8]. This new trend seems to be alarming in light of recent findings showing an early onset of CRC among 20–49-year age groups [12–15], which could lead to an increase in the burden of this disease in the coming 15–20 years. Thus, the evidence shows that the incidence rate of newly diagnosed cases of CRC is estimated to double in the coming decade if predictive, preventive, and personalized medicine approaches are not adopted as diagnostic and treatment modalities [16,17]. The five-year survival of CRC seems to depend on the stage of the disease; for example, the chances of survival with stage I CRC is 90% as compared with stage IV CRC patients, where it hardly reaches 10% [18]. Of note, the year overall survival (OS) of Saudi patients based on tumor stage was lower than that of internationally reported data on survival rates among CRC [19].

In this scenario, there is an urgent need to place and implement better early stage screening or detection and reliable diagnostic tools of prognostic significance in order to diagnose the disease early as well as predict chemotherapeutic response outcomes effectively. In this endeavor, a deep understanding of colorectal cancer biology has led to the establishment of genomic instability and the mitogen-activated protein kinase (MAPK) signaling pathway as a key orchestrator in the processes of disease induction, progression, and ultimately poor prognosis [20–23]. However, genomic instability plays a key role in CRC, as shown by three different independent research groups, who have established mainly microsatellite instability (MSI), CpG island methylator phenotype (CIMP), and chromosomal instability (CIN), which are major pathogenic mechanisms responsible for 80–85% of all CRC cases [20]. Notably, such aberrations in gene levels within these types of pathways affect cell proliferation and survival, for example, *WNT, MAPK/PI3K, TGFβ, TP53*, and mutations in various genes such as *c-MYC, BRAF, PIK3CA, PTEN, SMAD2*, and *SMAD4*, and more importantly RAS. Evidence suggests that three human RAS genes (*KRAS, NRAS*, and *HRAS*) are the key oncogenes mutated at high frequency in human cancer, including 90% in pancreatic cancer, 35% in lung cancer, and 45% in colon cancer. The high prevalence of mutations in RAS attracts it as a key target for cancer drug development and prognostic significance [21].

Among RAS genes, *KRAS* (*KRAS proto-oncogene, GTPase*; HGNC:6407) is the predominant and widely mutated isoform reported in the lung, pancreas, and CRC [21]. To date, several mutations have been reported as contributing factors in the development of CRC; for example, oncogenic rat sarcoma virus (RAS) mutations have been found to be prevalent in up to 50% of sporadic CRCs and 50% of colonic adenomas greater than 1 cm in length as compared to smaller adenomas, which are rarely seen [24,25]. The RAS gene normally encodes a group of GTPases that regulate cellular signal transduction, but the occurrence of deleterious mutations in the RAS gene alters the protein activity, resulting in a decrease in GTPase activity. In this way, *KRAS* acquires oncogenic properties, and such alterations lead to abrogate GTP hydrolysis by GTPase, resulting in a structurally active GTP-bound protein and hence acts as a constant growth stimulus [26,27]. The above results suggest that the gain of potential mutations in the RAS proto-oncogene activates its oncogenic potential and consequently initiates the tumor formation process [22,23]. Notably, among the three members of the RAS gene (*KRAS, HRAS*, and *NRAS*) superfamily, nearly 40% of mutations reported in the *KRAS* gene belong to CRCs [23]. Furthermore, the majority of *KRAS* mutations have been reported in exon-2 of the *KRAS* gene, whereas only 1% in exon-3 and 4% in exon-4 mutations are found in CRCs [28]. Moreover, the point mutations of *KRAS* were most frequently observed in codon 12 as compared to codons 13 and 61, in which the frequency of the mutation was found to be lower in CRCs [29]. Of note, mutations in *KRAS* codon 12 or 13 (exon 2) are considered as a major driving force in contributing oncogenic potential to *KRAS*, as evidenced by the fact that these mutations constitute almost 90% of all *KRAS* mutations in CRC [18,30–32]. The prognostic significance of *KRAS* mutations as a useful biomarker in predicting susceptibility, drug response outcomes, and survival of CRC patients has not been extensively investigated, particularly its prognostic value in metastatic CRC (mCRC).

Hence, from this perspective, the main objective of our study is to establish the mutational spectrum of the *KRAS* gene in the western region of Saudi Arabia in CRC patients to better understand the molecular etiology of CRC in this region, where very few studies have been conducted previously.

## Materials & methods

### Sample collection

We collected 80 colorectal cancer tumor tissue samples from patients undergoing surgery at King Abdulaziz University Hospital, Jeddah. The study was approved by the ethical committee of the Center of Excellence in Genomic Medicine Research (CEGMR). Written informed consent was obtained from all participants at the time of sample collection. Complete clinical information was obtained from patients, clinicians, and medical records or history of patient files in hospital records. The tumor samples were immediately transported to CEGMR and stored in the biobank under appropriate conditions until the start of molecular work.

### DNA extraction

DNA was extracted from all the tumor tissues of patients using the DNeasy® Blood & Tissue Kit (QIAGEN) according to the manufacturer’s protocol. The quality and quantity of the purified DNA were also checked using spectrophotometry and agarose gel electrophoresis. To estimate the quantity and asses quality, we used NanoDrop® ND-2000 from Thermo Scientific Inc. The integrity and quality of DNA were also assessed by running the extracted DNA in a 1% agarose gel in a horizontal gel electrophoresis tank apparatus with ethidium bromide staining and visualized under a UV illuminator.

### Sanger’s sequencing

Exon 2 of the *KRAS* gene was amplified by polymerase chain reaction using Forward 5ʹ-AACCTTATGTGTGACATGTTC-3ʹ and Reverse 5ʹ-TCCTGCACCAGTAATATGC-3ʹ primers [33]. The amplified product was then sequenced using Sanger’s method on an ABI 3730xl sequencer using the Big Dye Terminator®. BioEdit Sequence Alignment Editor Version 7.2.5 was used to generate sequencing peaks and interpret the results.

#### *KRAS* three-dimensional structure and mutation analysis

The homology model of the *KRAS* protein was designed using the Swiss model automated homology modeling platform to generate a three-dimensional (3D) structure [34]. The FASTA sequence of *KRAS* was downloaded from the UniProt Knowledgebase (UniProt ID: PO1116, conforming to a 188 amino acid (aa) transcript (NCBI Nucleotide ID: NM_004985.5; Ensembl Protein ID: ENSP00000308495.3) [35]. A template search with BLAST and HHblits was performed against the SWISS-MODEL Template Library (SMTL, last update: 2021–05-19, last included PDB release: 2021–05-14) [36–38]. Of the 906 templates identified in SMTL, 28 were found to be suitable for building *KRAS* homology models. The *KRAS* homology model was generated based on target-template alignment using ProMod3 [39] and the per-residue and global model quality was evaluated using QMEAN [39]. The MolProbity score was assessed for the *KRAS* homology model using a previously described method [40]. Ramachandran plots were generated using MolProbity (version 4.4) [41,42].

#### *KRAS* mutation analysis

The impact of the novel missense mutation (SER17ARG) identified in our study listed in Table 1 on the *KRAS* homology model was evaluated by investigating the structural features available in the Missense3D algorithm, such as clash, breakage of disulfide bonds, buried proline introduction, buried charge switch, allowed phi/psi, secondary structure alteration, buried/exposed, switch, buried charge replacement, buried glycine replacement, buried H-bond breakage, introduction of buried hydrophilic amino acids, introduction of buried charge crest replacement, glycine, and replacement in a bend, buried salt bridge breakage, and cavity modification [43].

**Table 1.** Nucleotides and amino acid changes in *KRAS* gene in CRC patients

S. No	No. of patients	Nucleotide change	Protein change	Reported or Novel
**Codon 12**				
1	1	c. 34 G>T	p. Gly12Cys	Reported
2	2	c. 34 G>C	p. Gly12Arg	Reported
3	2	c. 35 G>C	p. Gly12Ala	Reported
4	5	c. 35 G>T	p. Gly12Val	Reported
5	6	c. 35 G>A	p. Gly12Asp	Reported
**Codon 13**				
6	1	c. 37 G>T	p. Gly13Cys	Reported
7	2	c. 38 G>A	p. Gly13Asp	Reported
**Codon 17**				
8	4	c. 51 T>A	p. Ser17Arg	Novel (this study)
**Codon 31**				
9	3	c. 91 G>A	p. Glu31Lys	Reported in only 1 patient (ultra-rare)

## Results

**Figure 1.**
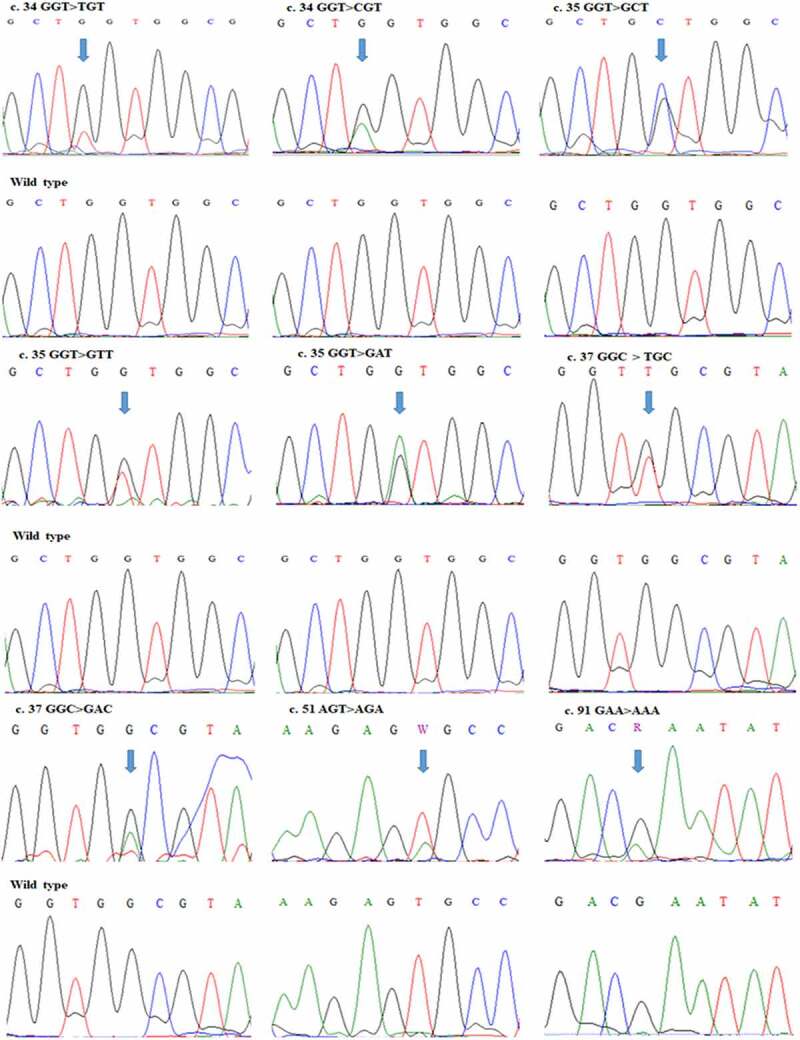
Chromatograms showing mutations in *KRAS* gene in representative patients

The current research focuses on the establishment of *KRAS* gene mutation spectrum in the kingdom of Saudi Arabia. Therefore to achieve the goal we recruited 80 CRC patients and performed *KRAS* gene sequencing from tumor samples. In this study, we identified *KRAS* gene mutations in 26 patients with CRC. The results are summarized in Table 1, and representative chromatograms are presented in Figure 1. Sanger sequencing revealed nine different types of mutations in exon 2, where the respective codons were 12, 13, 17, and 31. Most of the mutations were recorded in codon 12 of the gene, where we found five different types of mutations in 16 patients. Codon 13 harbored two mutations in three patients. Interestingly, we found a novel mutation in codon 17 of the *KRAS* gene in four patients. In codon 31 we found a very rare mutation in three patients.

The characteristics regarding age, gender, tumor location, pre and post-operative carcino-embryonic antigen (CEA), number of surgeries and response to treatment for patients underlying *KRAS* gene mutations are mentioned in Table 2. CEA levels above 3 ng/ml are considered to be elevated and taken as abnormal. The increased values of CEA are indicative of presence of many types of cancer particularly of CRC. After surgical removal and treatment of the tumors in CRC patients these values seemed to decrease significantly as shown in Table 2. CEA may help in checking the recurrence in asymptomatic patient and it is also used in detection of liver metastasis as an early indicator in CRC [44]. In this study cohort, most of the patients seen reduced levels of CEA after surgical removal of tumor. Only three patients are shown to have higher values of CEA after surgery and treatment and may indicate the metastasis of the cancer in other organs. Sometimes the CEA levels are also raised in some non-cancerous conditions like liver disease, ulcerative colitis, chronic bronchitis, pulmonary emphysema and inflammatory bowel disease [45].

**Table 2.** Characteristics of CRC patients with *KRAS* mutations

**S. No**	**Sample ID**	**Mutation**	**Age**	**Gender**	**Tumor Location**	**Preoperative CEA**	**Postoperative CEA**	**Surgery**	Response to Treatment*
1	CR-576	p. G12C	55	Female	Right	50	0.3	2	1
2	CR-2090	p. G12R	60	Male	Left	5.2	0.32	2	2
3	CR-934	p. G12R	28	Female	Left	3.5	1.6	2	2
4	CR-4690	p. G12A	23	Female	Left	4.2	0.2	2	3
5	CR-4688	p. G12A	23	Female	Right	3.4	0.4	1	1
6	CR-926	p. G12V	65	Female	Right	6.6	1.13	1	1
7	CR-104	p. G12V	51	Male	Left	20.5	2.51	2	1
8	CR-196	p. G12V	62	Male	Left	8.9	2.7	2	2
9	CR-1039	p. G12V	58	Male	Right	13.42	0.7	3	2
10	CR-220	p. G12V	42	Female	Left	9.8	3.8	2	2
11	CR-5771	p. G12D	47	Male	Left	6.9	1.7	1	1
12	CR-218	p. G12D	70	Female	Right	2.4	0.2	2	2
13	CR-384	p. G12D	82	Male	Rectal	12.5	5	2	1
14	CR-230	p. G12D	63	Male	Left	45	130	3	3
15	CR-234	p. G12D	57	Female	Rectal	1.4	0.6	1	1
16	CR-477	p. G12D	45	Male	Right	14.8	10.7	2	2
17	CR-1080	p. G13C	59	Male	Left	11.61	1.4	2	3
18	CR-72	p. G13D	31	Female	Right	1.99	2.07	2	1
19	CR-117	p. G13D	52	Female	Left	127	5.5	2	2
20	CR-692	p. S17R	66	Male	Right	10.6	1.9	1	2
21	CR-106	p. S17R	54	Male	Left	1.26	0.5	2	1
22	CR-163	p. S17R	75	Female	Left	127	5.5	2	2
23	CR-210	p. S17R	50	Male	Rectal	10.6	1.9	2	2
24	CR-961	p. E31K	65	Female	Right	2.8	3.2	2	2
25	CR-601	p. E31K	35	Male	Left	14.4	0.9	1	1
26	CR-181	p. E31K	73	Male	Right	6.05	2.52	2	2

*(1 = Complete response, 2 = Partial response, 3 = No response)

To create a homology model for the wild-type (WT) *KRAS* protein, the target sequence obtained from UniProt was searched using BLAST against the primary amino acid sequence contained in the SMTL [46]. An initial HHblits profile was built [37] and searched against all profiles of the SMTL. We found 28 suitable template homology models for *KRAS*. The KRAF homology model was built using the top protein template 4dst.1. A GTPase *Kras* Isoform 2B.

**Figure 2.**
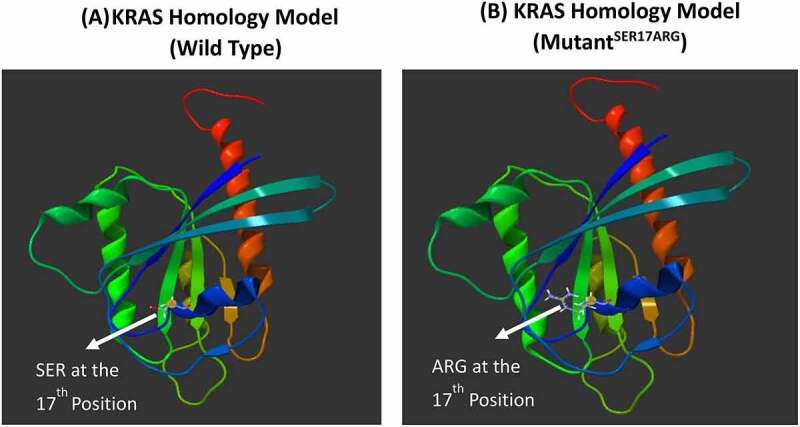
*KRAS* homology model of wild and mutated proteins. *KRAS* model was build using Swiss Model automated homology modeling platform based on the FASTA sequence downloaded from the UniProt Knowledgebase: (a) *KRAS* homology model of wild type (WT) protein showing the Ser at the 17^th^ position (b) *KRAS* homology model of the novel missense mutation identified in our study showing the substitution of Ser with Arg at the 17^th^ position

The 3D homology model of *KRAS* was tested using the novel mutation of serine replaced by arginine at position 17 using the missense 3D algorithm to assess the impact of this mutation (Figure 2). No damage was predicted in the *KRAS* structure by Missense 3D based on the structural parameters evaluated, such as F01: disulfide breakage: N|F02: buried Pro introduced: N|F03:Clash: N|F04:Buried hydrophilic introduced: N|F05:Buried charge introduced: N|F06:Secondary structure altered: N|F07:Buried charge switch: N|F08:Disallowed phi/psi: N|F09:Buried charge replaced: N|F10:Buried Gly replaced: N|F11:Buried H-bond breakage: N|F12:Buried salt bridge breakage: N|F13:Cavity altered: N|F14: Buried/exposed switch: N|F15:Cis pro replaced: N|F16:Gly in a bend: N| (Figure 2).

### General glycine proline preproline

**Figure 3.**
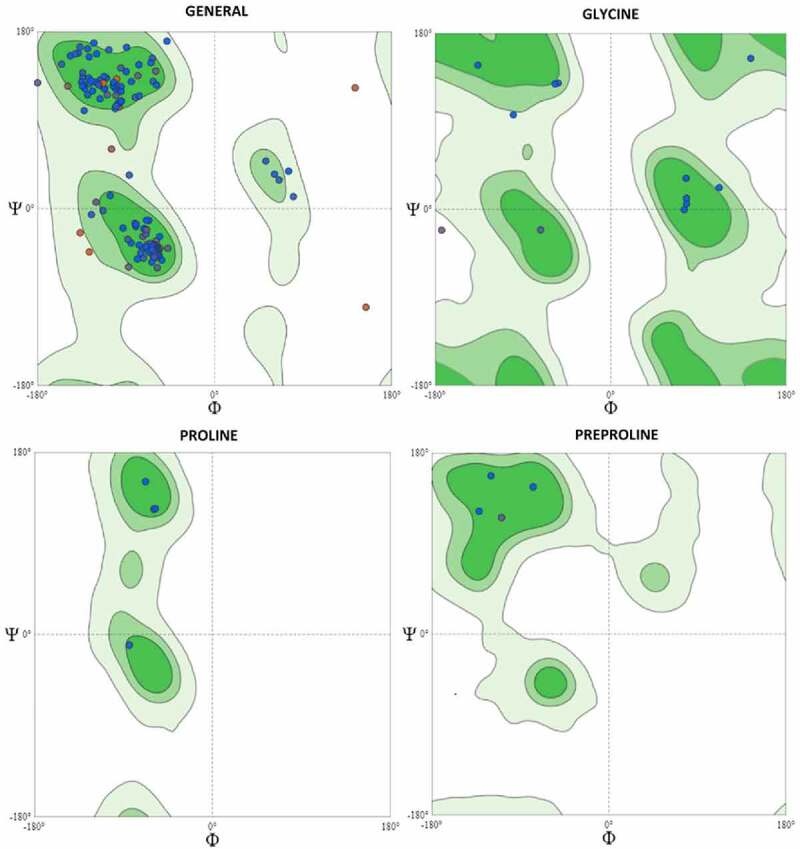
**Ramachandran Plots for *KRAS* Homology Model (Wild Type)**. The Ramachandran plots of the *KRAS* homology model showed that 93.22% of residues were in the favored region, 2.26% were in the outlier region, rotamer outliers were 3.80%, C-beta deviations were 4, bad bonds were 0 out of 1453, bad angles were 34 out of 1951 and Cis Non-Proline 2/174

In addition, the *KRAS* homology model has a MolProbity score of 1.86 with a Clash Score of 2.07. The Ramachandran plot of the *KRAS* homology model showed that 93.22% of residues were in the favored region, 2.26% were in the outlier region, rotamer outliers were 3.80%, C-beta deviations were 4, bad bonds were 0 out of 1453, bad angles were 34 out of 1951, and Cis Non-Proline 2/174 (Figure 3).

### Discussion

In recent times, the research and identification of variants has become increasingly important to evaluate and correlate the drug response in personalized medicine and opting for chemotherapeutic strategies. The recent model for CRC is the multistep genetic process proposed by Fearon and Vogelstein and is considered to be the standard for carcinogenesis in solid tumor progression [47]. In the initiation of carcinogenesis, adenomatous polyposis (APC), a tumor suppressor gene, inactivates and leads to mutational activation of the *KRAS* gene. The subsequent mutational changes in the TP53, PIK3CA, and TGF-β pathways are responsible for the malignant transformation of tumors [48]. Seven distinct mutations were considered to be vital in this model, while recent progress in molecular sequencing techniques has identified more than 80 mutated genes underlying a single CRC. However, only 15 genetic mutations have recently been considered the main drivers of carcinogenesis [49,50].

Oncogenic mutations in the *KRAS* gene play a central role in these models because of their important role as well as their early involvement in tumorigenesis. The majority of mutations in the *KRAS* gene have been reported previously in codons 12 and 13 of exon 2 [51,52]. Therefore, we decided to sequence exon 2 of the *KRAS* gene in our study in the Saudi population to determine the landscape of mutations in this region.

To define the spectrum of mutations in the *KRAS* gene in the Saudi population, we checked mutations in 80 CRC tumor tissues. The study revealed mutations in 26 patients (32.5%) in four different codons of exon 2, that is, 12, 13, 17, and 31. Most of the mutations were identified in codon 12 in 16 patients, or 61.5% of all mutations. Previous studies have also shown that these mutations in codon 12 are the most common in CRC [53]. At codon 12, glycine was substituted by aspartate (G12D) in 6 patients (23%). G12D is a hot spot and accounts for the majority of *KRAS* mutations [52]. The second most common mutation was glycine substituted by valine (G12V) in five patients (19.2%). This mutation has also been reported in the literature to be the second most common mutation [54]. However, some studies have also reported this as the most common variation in their studies [55]. The remaining three mutations were glycine substituted by arginine (G12R in two patients), glycine substituted by alanine (G12A in two patients), and glycine substituted by cysteine (G12C in 1 patient). The presence of glycine at position 12 seems to be very important for proper functioning of the *KRAS* gene, and disruption or replacement of this amino acid with other disrupts or creates structural damage along with failure in function efficiency [54].

In codon 13, glycine was replaced by aspartate (G13D) in two patients and glycine substituted by cysteine (G13C) in one patient sample. Codon 13 is considered to be the second most hot spot for mutations, as many of the studies reported the second highest mutation after codon 12 [54,55].

In codon 17, we found mutations in four patients where serine was substituted with arginine (S17R). This mutation seems to be novel as no previous study described in the literature to the best of our knowledge. Therefore, this mutation may be very useful for the prognosis in Saudi patients. No structural changes were observed in the *KRAS* homology model due to the replacement of Ser by Arg (Figure 3). However, the cavity volume is exposed by 4.536 Å ^3 by this novel missense mutation, and there was no disulfide bond breakage since the WT residue was not Cys. Ser is exposed (RSA 24.6%) and Arg is exposed (RSA 16.5%), there is no clash, and disallowed phi/psi alert because the phi/psi angles are in the favored region for the wild-type and mutant residues (Figure 3). The local clash score for wild type is 15.13 and 18.62 for the mutant. The buried hydrophobic residue with a hydrophilic residue was not replaced, and buried uncharged residue alerts and switch alerts were not triggered. Ser is exposed neutral with RSA 24.6%, and the mutant residue Arg is exposed hydrophilic with RSA 16.5%. A wild-type salt bridge was detected between the OD1 atom of Asp 173 and the NZ atom of LYS 169 (distance: 2.480 Å). However, a salt bridge is also found in the mutant structure between the OD1 atom of Asp 173 and the NZ atom of LYS 169 (distance: 2.480 Å). The wild-type SER was exposed (RSA, 24.6%). The WT residue is not a Gly or cis-proline, and the substitution with Arg at the 17^th^ position does not alter the secondary structure ‘H’ (4-turn helix). Ser and Arg were exposed uncharged with RSA of 24.6% and 16.5%, respectively.

In codon 31, we found a mutation in three patients where glutamic acid was replaced by lysine (E31K). This mutation is very rare and has only been found in one of the previous studies in the Pakistani population and only in a single patient [56]. No further evidence of this mutation has been found in CRC, although this mutation has been previously reported in endometrioid carcinoma [57]. Therefore, our findings also confirm the presence of the mutation E31K in the Saudi population. We found this mutation in three Saudi patients.

## Conclusions

The main advantage of this study is the identification of variants in the *KRAS* gene associated with colorectal cancer in the western parts of Saudi Arabia. In this study, we discovered a novel mutation associated with CRC in the *KRAS* gene, S17R, in four Saudi patients. Moreover, we also discovered a very rare mutation, E31K, in three Saudi patients.

## Highlights

1. Frequency and mutational spectrum of *KRAS* gene in colorectal cancer patients.
2. Discovery of a novel mutation c.51G>A (S17R) in four patients.
3. Identification of a very rare mutation c.91G>A (E31K).

## Limitations and Future Recommendations

More studies with larger patient samples are needed to establish mutational spectrum in Saudi population. Further functional characterization of these mutations is needed to fully understand their role in the development and progression of CRC.
